# Is Your Color My Color? Dividing the Labor of the Stroop Task Between Co-actors

**DOI:** 10.3389/fpsyg.2018.01407

**Published:** 2018-08-06

**Authors:** Motonori Yamaguchi, Emma L. Clarke, Danny L. Egan

**Affiliations:** Department of Psychology, Edge Hill University, Ormskirk, United Kingdom

**Keywords:** joint performance, Stroop interference, semantic gradient, division of labor, co-representation

## Abstract

Performing a task with other actors involves two opposing forces, division of labor between co-acting individuals and integration of divided parts of the task into a shared mental representation (co-representation). Previous studies have focused primarily on the integration of task representations and limited attention has paid to the division of labor. The present study devised a test of the integration and the division in a joint task setting. A joint version of the Stroop task was developed, in which pairs of actors were assigned different sets of target colors. If the actors integrate their co-actor’s task, the colors assigned to their co-actor should be represented as if they were the actor’s own target colors; the Stroop effect should be as large when distractor color words denote their co-actor’s target colors as when these words denote the actor’s own target colors. If the actors divide the labor of the Stroop task, the colors assigned to their partner should be represented as non-target colors; the Stroop effect should be smaller when the distractor color words denote the co-actor’s target colors than when these words denote the actor’s own target colors. The results of response time did not provide clear support for either position, while those of response accuracy supported the division of labor. Possible cognitive mechanisms that support the division of labor and the integration of task representation are discussed.

## Introduction

Performing a single task jointly with other actors provides an opportunity to divide the labor of the task between co-acting individuals. The division of labor reduces the workloads of the individuals and allows them to focus more efforts on part of the task to which they are assigned. This gives rise to an advantage of group performance (Wegner, 1986). A joint task may also require coordinating actions of the co-acting individuals, which enables collective efforts to accomplish work that is greater than what might be achieved by each of the individuals alone (e.g., moving a heavy furniture). Coordinating actions with co-actors requires monitoring actions of the others, and this is made possible by integrating others’ task contexts into the actor’s own task representation (Knoblich and Sebanz, 2006). Nevertheless, coordination requires each actor to monitor their co-actors at a given moment, which would impose an additional workload and make the actors’ actions interdependent, imposing additional constraints on their own actions. Therefore, the division of labor and the coordination of actions are two opposing forces that need to be balanced for a successful completion of a joint task (see Moreland, 1999, for a similar idea in organizational contexts).

Studies of joint action have focused on the integration of task representations, or *task co-representation* (Sebanz et al., 2003; Knoblich et al., 2011). The notion of co-representation has been supported by the findings in the *joint Simon task* (Sebanz et al., 2003). The Simon task is a choice-reaction task in which an individual actor responds to non-spatial attributes of stimuli (e.g., colors) by pressing response keys on the left or right. The actor is asked to ignore the stimulus location, but responses are still faster and more accurate when stimulus and response locations correspond than when they do not, yielding the Simon effect. The actors in the joint Simon task divide the labor of the Simon task in such a way that one actor responds to one stimulus type (e.g., red circles) by pressing one response key (e.g., on the left) and the other actor respond to the other stimulus type (green circles) by pressing the other key (on the right). This is essentially a go/nogo task that only requires each actor to respond to stimuli on some trials and withhold responding on other trials. When the same go/nogo Simon task is performed by a single actor, no Simon effect is obtained (Hommel, 1996), because the spatial attribute is no longer relevant to represent the response, eliminating the spatial correspondence between stimulus and response. With two actors performing together, the joint Simon task still produces the Simon effect, implying that the spatial attribute is used to represent the responses. This finding has led researchers to suggest that co-acting individuals not only represent their own part of the task but also integrate their co-actor’s part into their own task representation. Such a joint task representation completes the entire picture of the Simon task. A strong version of this co-representation account suggests that the actors represent their co-actor’s actions as if these actions were on the actors’ own command (Knoblich and Sebanz, 2006).

Task co-representation would enable a better coordination of actions between co-acting individuals. However, by considering their co-actor’s actions, task co-representation can also cause additional cognitive conflicts between the actor’s own action program and the action program representing their co-actor’s response that does not need to be executed (Sebanz et al., 2006). Nevertheless, there has also been evidence suggesting that co-acting individuals may not represent their co-actor’s part of the task or actions; instead, they represent their own actions with reference to their co-actor’s actions (Dolk et al., 2014). If so, the co-acting individuals do not necessarily monitor what their co-actor does or how their co-actor performs their part of the task, but they may simply be aware of the fact that they have divided the labor of a joint task with their co-actor. Previous findings support this position, showing that the actors in the joint Simon task monitor the proportion of compatible trials for their own part but not for their co-actor’s part (Yamaguchi et al., 2018) and that the actors in a joint task-switching setting do not monitor the task that their co-actor has performed on a preceding trial (Wenke et al., 2011; Yamaguchi et al., 2017b). Such task monitoring appears to occur under specific conditions (Dudarev and Hassin, 2016; Liefooghe, 2016; Yamaguchi et al., 2017a). Therefore, the actors may only represent limited aspects of the co-actor’s part of the task, and they divide the labor of the joint task, eliminating an additional burden monitoring their co-actor’s part of the task. The purpose of the present study was to devise another test of the division of labor and the integration of task representation in a join task setting.

### Joint Stroop Task

Previous studies suggest that actors do represent stimuli that occurred on the co-actor’s trials (Dolk et al., 2013; Eskenazi et al., 2013) and the action that the co-actor has made on a preceding trial (Welsh et al., 2005). The present study assessed whether stimuli (and, to some extent, responses) assigned to their co-actor are represented as part of the actor’s own task. To this end, a joint version of the Stroop task (Stroop, 1935) was utilized. The Stroop effect is one of the most robust interference phenomena that can easily be reproduced even under an uncontrolled environment, such as a college seminar room. It occurs when people try to name the colors of color words whose meanings are incongruent with the colors that they are meant to name (e.g., the word “BLUE” printed in red). The Stroop effect is often thought to involve a quintessential form of automaticity (e.g., LaBerge and Samuels, 1974; Posner and Snyder, 1975; MacLeod, 1992), but it has also been shown that the effect depends on a number of factors (e.g., Kahneman and Treisman, 1984; Moors and De Houwer, 2006). Importantly to the present study, the Stroop effect has been shown to depend on how the irrelevant word names are related to the target colors to which participants respond (Klein, 1964; Levin and Tzelgov, 2016). In particular, Stroop interference is largest when the task-irrelevant word names come from the set of the target colors, but it decreases when the task-irrelevant word names are from outside the set of the target colors. For instance, if the target colors were ‘red’ and ‘green,’ then the word ‘YELLOW’ would produce less interference than the word ‘RED.’ This finding has been known as *semantic gradient*. The present study used this finding to address the issue of what aspects of the co-actor’s task the actors represent when performing a task jointly with others.

**FIGURE 1:**
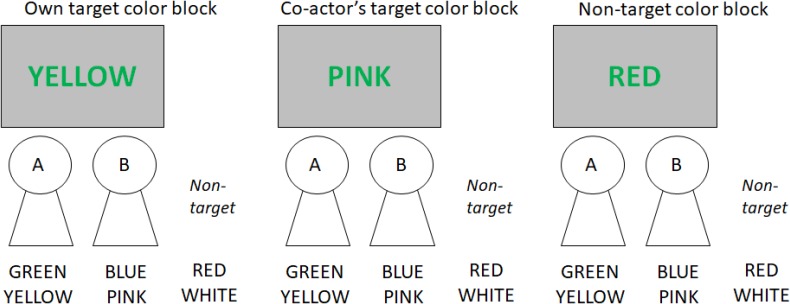
Examples of incompatible trials in the three block types. The examples are trials for which the color is green and Actor A should make a response. The color names under the actors A and B are the target colors assigned to the respective actors; the color names under “non-target” are non-target colors.

In the present version of the joint Stroop task, a pair of actors performed the Stroop task with a set of four target colors. Each actor responded to two of the four colors by pressing response keys. As Klein (1964) showed, the size of the Stroop effect should depend on how closely the word meanings are related to the target colors to be named, producing the semantic gradient of Stroop interference. Levin and Tzelgov (2016) recently showed that the semantic gradient occurs when different types of distractor words were presented in separate blocks but not when they were intermixed within a block. Consequently, the present study tested three types of blocks across which different types of irrelevant word names occurred (see **Figure 1** for examples).

In the first block, incompatible word meanings denoted the target color names assigned to the actor him- or herself (*own target color block*); in second block, incompatible word meanings denoted the target color names assigned to the co-actor (*co-actor’s target color block*); and in the third block, incompatible word meanings denoted the non-target color names that were not assigned to either actor (*non-target color block*). All of these blocks also included compatible trials for which the irrelevant word meanings denoted the names of the colors in which the words occurred. Based on Klein (1964) semantic gradient, it was expected that the Stroop effect would be larger in the own target color block than in the non-target color block. The main question was one of whether the Stroop effect in the co-actor’s target color block was similar to that in the own target color block or that in the non-target color block.

If the actors in the joint Stroop task co-represent their co-actor’s part of the task, then the actors should react to the co-actor’s target colors as if they were their own target colors. In this case, the Stroop effect in the co-actor’s target color block should be similar to the effect in the own target color block and should be larger than the effect in the non-target color block. Such outcomes would imply that actors integrated their co-actor’s target colors into their own set of target colors. If the actors do not co-represent co-actor’s part of the task, then the actors should not react to the co-actor’s target colors as if they were their own target colors. Thus, the Stroop effect in the co-actor’s target color block should be similar to the effect in the non-target color block and should be smaller than the effect in the own target color block. Such outcomes would imply that actors divided the labor of monitoring their own set of target colors from their co-actor’s target colors.

It is worth noting two recent studies that also used a joint version of the Stroop task (Demiral et al., 2016; Saunders et al., 2018). In the first study by Demiral et al. (2016), the main purpose was to compare individual and joint task conditions in terms of the ERP signal. An important finding was that the P3b component of ERP, which was thought to reflect a translation from stimulus to response, increased on nogo trials of the joint task (which was performed by the co-actor) as compared to nogo trials of the individual task. This suggested to the authors that actors ‘mapped stimuli onto the co-actor’s response’ even when these actors did not need to perform the task on the co-actor’s trials, consistent with the co-representation view. However, on nogo trials of the joint task in their experiment, the actors were required to report whether their co-actor’s response was correct by saying ‘yes’ or ‘no’ and by pressing a key in case the co-actor made an error. This requirement on nogo trials forced the actors to monitor their co-actor’s trials and determine what response the co-actor should be making. Thus, monitoring of the co-actor’s responses was built in the task, and it is not surprising that the actors had to perform the co-actor’s trials (mentally) in such a situation. The present study used a version of the Stroop task without this additional requirement, and it would be the actor’s spontaneous choice if they co-represent their co-actor’s target colors as part of their own task representation. Hence, the present design provided a stronger test of task co-representation in a joint Stroop task than Demiral et al. (2016). The second study by Saunders et al. (2018) study was similar to the present study, but a main difference was that all types of distractor words were intermixed within a single block in that study. The present study separated the three types of distractor words (own target color, co-actor’s target color, and non-target color) into different blocks because Levin and Tzelgov (2016) reported that the semantic gradient was observed only when different distractor words were separated between blocks. As the semantic gradient played a central role in formulating the hypotheses, this is an important methodological feature of the present study. Also, the present study involved two alternative responses per actor, rather than one response per actor in Saunders et al. (2018) study. These differences could determine whether the actors co-represent in a joint task, so it is important to assess whether the results of the present study would deviate from those reported by Saunders et al. (2018).

The present study consisted of two experiments, which differed in two respects. First, the individual task in Experiment 1 consisted only of trials for which the target colors were always from the actor’s own target colors, so that the actors responded on all trials (i.e., all trials were go trials). The individual task in Experiment 2 consisted of trials for which the target colors were either from the actor’s own target colors or their co-actor’s target colors, so that the actors responded on half of the trials (go trials) and withheld responding on the other half (nogo trials). The number of trials was the same for the two experiments, but these procedural differences meant that the number of go trials that each actor performed between the individual and joint tasks was the same in Experiment 1, whereas the number of go and nogo trials that occurred in a block between the individual and joint tasks was the same in Experiment 2. Second, the sample size was nearly doubled in Experiment 2 to examine whether the main results of Experiment 1 were replicated.

## Experiment 1

### Method

#### Participants

Thirty-two participants participated in the present experiment (21 females; mean age = 19.42, *SD* = 1.50, range = 18–21). Twenty-four participants were originally recruited, and eight participants were added later as per the suggestion from a reviewer to match the sample size in Saunders et al. (2018) Experiment 1. All participants were recruited from the Edge Hill University community in pairs. With the current design, a statistical power of at least 0.95 is achieved for a medium effect size if the sample size is 18 or above. Each participant in a pair received £3 for participation. All participants reported having normal color vision and normal or corrected-to-normal visual acuity. They were naïve as to the purpose of the experiment. The present study followed the recommendations of the British Psychological Society Code of Ethics. All subjects gave written informed consent in accordance with the Declaration of Helsinki. The protocol was approved by the the Departmental Research Ethics Committee of the Psychology Department at Edge Hill University.

#### Apparatus, Stimuli, and Procedure

The apparatus consisted of a 23-in widescreen monitor and a personal computer. Stimuli were six color words (GREEN, RED, BLUE, YELLOW, PINK, and WHITE), presented in one of the same six colors against a gray background. The stimuli were presented in the Arial font at the 60-pt font size. Responses were registered by pressing keys on a QWERTY desktop keyboard.

The experiment was conducted individually for each pair in a cubicle under normal fluorescent lighting. Participants read on-screen instructions, and the participant who sat on the left side (Actor A) placed their left and right index fingers on the ‘z’ and ‘c’ keys, respectively, and the participant who sat on the right side (Actor B) placed their left and right index fingers on the ‘1’ and ‘3’ keys on the numerical keypad. For each pair, four colors were chosen and assigned randomly to the four keys (see **Figure 1** for an example). These colors appeared as the target color to which participants responded, and the remaining two colors were used only as irrelevant word meanings.

Each pair performed two phases, the *individual task phase* and the *joint task phase*. In the individual task phase, each participant performed trials alone while the co-actor remained inactive. For each participant, there was one block of 12 practice trials and three blocks of 64 test trials each. Each test block consisted of 32 compatible trials for which the task-irrelevant word meaning was the same as the target color, and 32 incompatible trials for which the task-irrelevant word meaning was different from the target color. The target colors were either of the two colors assigned to the actor. There were three types of blocks in which the irrelevant word meanings on incompatible trials were manipulated (**Figure 1**). In the first type of blocks (*own target color block*), the task-irrelevant word meanings were colors assigned to the actor who was performing the task. In the second type of blocks (*co-actor’s target color block*), the task-irrelevant word meanings were colors assigned to the co-actor who was not performing the task. In the third type of blocks (*non-target color block*), the task-irrelevant word meanings were colors that were not assigned to either participant in the pair. In each of the test blocks, there were two possible color words on incompatible trials that occurred in an equal frequency and in a random order; the two target colors also occurred in an equal number of trials. In the practice block, all types of task-irrelevant word meanings could occur, and trials were randomly chosen without replacement. The order of the test blocks was permuted to counterbalance across pairs, and it was maintained for the two actors.

In the joint task phase, both participants performed trials, and which actor responded on a given trial depended on the target color. The joint task phase was similar to the individual task phase; it consisted of one block of 12 practice trials and two cycles of three blocks of 64 test trials each (six test blocks in total). Two of the three test blocks in each cycle used the colors assigned to one of the actors, so a block was the own target color block for one actor and it was the co-actor’s target color block for the other actor. The remaining block was the non-target color block for both actors. The order of the test blocks was the same in the two cycles.

Each trial started with a fixation cross at the screen center for 500 ms, followed by a 500-ms blank display. A word appeared at the center for 1,200 ms unless a response was made before the deadline. A feedback message was presented for 500 ms. The message was “Correct!” for the correct response, “Error” for an incorrect response, “Not your turn!” for a response by a wrong actor, and “Faster!” when there was no response within the 1,200-ms response window. Another 500-ms blank display was presented before the next trial. In the joint task, half of the trials in each block were assigned to one actor, and other trials to the other actor. In the individual task, all trials were assigned to one actor, and the other actor remained silent. Response time (RT) was the interval between word onset and a keypress.

### Results

**FIGURE 2:**
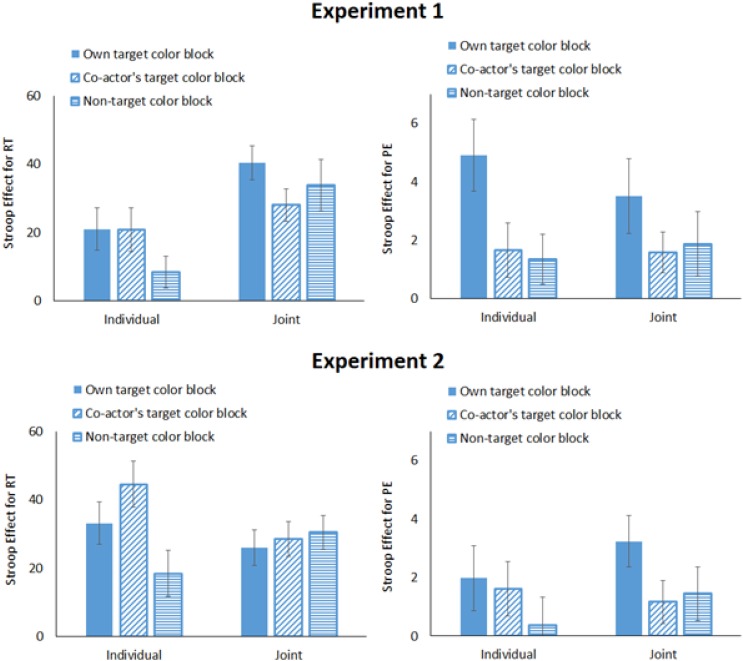
The Stroop effect for the individual and joint tasks in Experiments 1 and 2 (RT, response time; PE, percentages of error trials; and error bars represent one standard error of the mean).

Trials were discarded if RT was less than 200 ms, a wrong actor responded, or no response was registered within the 1,200-ms time window (1.23% of all trials). Mean RT and percentage errors (PEs) are summarized in **Table 1**. The Stroop effects are shown in **Figure 2**. RT and PE were submitted to 2 (Task Condition: joint vs. individual) × 3 (Block Type: own target color vs. co-actor’s target color vs. non-target color) × 2 (Stimulus Compatibility: compatible vs. incompatible) ANOVAs (see **Table 2**).

**Table 1:** Mean response time (ms) and percentage error (PE; values in the parentheses are standard errors of the mean) in Experiment 1.

Task Condition	Block Type	Compatible		Incompatible	
		*RT*			
Individual	Own color	414	(10.00)	435	(12.05)
	Co-actor’s color	419	(11.47)	440	(12.69)
	Other color	410	(9.53)	418	(10.97)
Joint	Own color	478	(11.80)	518	(14.39)
	Co-actor’s color	473	(10.73)	501	(12.15)
	Other color	468	(11.46)	502	(13.71)

		***PE***			
Individual	Own color	2.30	(0.57)	7.20	(1.41)
	Co-actor’s color	3.39	(0.86)	5.06	(1.12)
	Other color	2.79	(0.68)	4.14	(0.70)
Joint	Own color	4.49	(0.88)	8.00	(1.21)
	Co-actor’s color	3.28	(0.80)	4.86	(1.00)
	Other color	3.57	(1.09)	5.45	(0.96)

**Table 2:** The results of ANOVAs on response time and percentage error in Experiment 1.

Factors	*df*	*MSE*	*F*	*p*	$\eta_{\text{p}}^{2}$
	***Response time***				
Task Condition (TC)	**1, 31**	**4,963.98**	**87.42**	**<0.001**	**0.738**
Block Type (BT)	2, 62	2,194.31	2.24	0.115	0.067
Stimulus Compatibility (SC)	**1, 31**	**869.31**	**71.29**	**<0.001**	**0.697**
TC × BT	2, 62	1,445.59	1.66	0.199	0.051
TC × SC	**1, 31**	**707.57**	**10.17**	**0.003**	**0.247**
BT × SC	2, 62	381.40	1.97	0.148	0.060
TC × BT × SC	2, 62	474.79	1.45	0.243	0.045

	***Percentage error***				
TC	1, 31	39.27	1.55	0.223	0.048
BT	**2, 62**	**23.27**	**3.79**	**0.028**	**0.109**
SC	**1, 31**	**30.06**	**19.67**	**<0.001**	**0.388**
TC × BT	2, 62	13.42	1.74	0.184	0.053
TC × SC	1, 31	10.86	<1	0.638	0.007
BT × SC	**2, 62**	**16.81**	**4.26**	**0.019**	**0.121**
TC × BT × SC	2, 62	13.97	<1	0.577	0.018

*Effects in bold are significant at α = 0.05.*

Response time revealed main effects of Task Condition (*M*s = 423 ms for individual task and 490 ms for joint task) and of Stimulus Compatibility (*M*s = 444 ms for compatible trials and 469 ms for incompatible trials), and their interaction, which indicated that the Stroop effect was smaller for the individual task (*M* = 17 ms) than for the joint task (*M* = 34 ms). In the individual task, the Stroop effect was 21 ms for the own color block, 21 ms for the co-actor’s color block, and 8 ms for the non-target color block; in the joint task, the Stroop effect was 40 ms for the own target color block, 28 ms for the co-actor’s target block, and 34 ms for the non-target color block.

Percentage error revealed main effects of Block Type (*M*s = 5.50% for own target color, 4.15% for co-actor’s target color, and 3.99% for non-target colors) and Stimulus Compatibility (*M*s = 3.30% for compatible trials and 5.78% for incompatible trials). There was also a significant interaction between Block Type and Stimulus Compatibility. The Stroop effect tended to be larger for the own target color block (*M* = 4.21%) than for the co-actor’s target color block (*M* = 1.62%) but not for the non-target color block (*M* = 1.62%).

### Discussion

The Stroop effect was obtained in the present experiment. In RT, the Stroop effect was only numerically smaller for the non-target colors than the other colors in the individual task, and it was only numerically larger for the own target colors than the co-actor’s target or non-target colors in the joint task; however, none of these differences were supported statistically. These results provided little evidence for the semantic gradient that would have been expected if colors were represented differently according to whether they belong to the actor’s target set. Consequently, the RT data supported neither the division of labor or the integration of task representations. In PE, the Stroop effect depended on the block type, yielding a larger Stroop effect for the own target colors than the co-actor’s color target or non-target colors. These results are consistent with the division of labor, that is, when the co-actor’s target colors were represented as if they were non-targets.

As noted by Saunders et al. (2018), the discrepancy between RT and PE may reflect the possibility that there are two sources of the Stroop effect, stimulus recognition and response selection, and these measures may be sensitive to different processes. The Stroop effect could occur in stimulus recognition due to stimulus conflict, a conflict between the color name and an incongruent word meaning. The Stroop effect could occur in response selection due to response conflict, a conflict between the response that the color name indicates and the response that the incongruent word meaning indicates. The joint task eliminated response conflict in the co-actor’s target color block because the actors were never required to make the responses assigned to the co-actor’s target colors. Thus, the Stroop effect for the own target colors could involve stimulus conflict and response conflict, whereas the Stroop effect for the co-actor’s target colors could involve stimulus conflict but not response conflict. If this is the case, the RT results would imply that the Stroop effect in RT only reflected stimulus conflict and that all types of colors are represented similarly. The PE results would then imply that the Stroop effect in PE reflected both stimulus conflict and response conflict. Although these findings are consistent mostly with Saunders et al. (2018) study, the outcomes may depend on the use of manual responses as in the present study and Saunders et al. (2018). It would be interesting to see if the same results are obtained with vocal responses as in Demiral et al. (2016) study.

Another interesting outcome of the present experiment was that, in RT, the Stroop effect was larger for the joint task than for the individual task. Apart from the fact that the task was performed by one actor or two actors, a difference between the individual and joint task settings in the present experiment is that the target colors in the individual task were always the actor’s own target colors (i.e., go trials), whereas the target colors in the joint task were either the actor’s own target colors (go trials) or the co-actor’s target colors (nogo trials). The additional requirement of withholding responses on the co-actor’s trials might have increased the task demand and reduced attention that the actors could devote to their own trials. In fact, responses were generally slower in the joint task than in the individual task. The actors might have exercised stronger proactive control in the individual task than in the joint task, reducing the Stroop effect in the former case.

In Experiment 2, this difference between the individual and joint tasks was excluded, so that the actors were now presented with their own target colors on half of the trials and the co-actors’ target colors on the other half in the individual and joint tasks. The sample size was also nearly doubled to examine whether the lack of the differences among the three blocks types in RT merely reflected low statistical power in Experiment 1.

## Experiment 2

### Method

#### Participants

A new group of 62 participants (49 females; mean age = 21.23, *SD* = 3.14, range = 18–43) were recruited from the same university community.

#### Apparatus, Stimuli, and Procedure

The apparatus and stimuli were the same as those used in Experiment 1. The procedure closely followed that of Experiment 1, with the following changes in the individual task phase. In the individual task, the color words appeared in one of the four colors, two target colors of the actor and two target colors of the co-actor. On half of the trials, the stimuli were in the actor’s target color to which the actor responded (go trials); on the other half, the stimuli were in the co-actor’s target color with which the actor withheld responding to (nogo trials). Each of these target colors occurred equally frequently, and half of the trials were compatible trials and the other half were incompatible trials. Each test block consisted of 64 trials as in Experiment 1.

### Results

Trials were filtered in the same manner as in Experiment 1 (2.18%). Mean RT and PE are summarized in **Table 3**, and the Stroop effect is shown in **Figure 2**. RT and PE were submitted to 2 (Task Condition: joint vs. individual) × 3 (Block Type: own target color vs. co-actor’s target color vs. non-target color) × 2 (Stimulus Compatibility: compatible vs. incompatible) ANOVAs (**Table 4**).

**Table 3:** Mean response time (ms) and percentage error (PE; values in the parentheses are standard errors of the mean) in Experiment 2.

Task Condition	Block Type	Compatible		Incompatible	
		*RT*			
Individual	Own color	540	(8.91)	573	(10.14)
	Co-actor’s color	521	(9.53)	566	(11.17)
	Other color	550	(11.64)	569	(12.56)
Joint	Own color	519	(8.79)	545	(9.52)
	Co-actor’s color	508	(8.27)	537	(10.25)
	Other color	508	(10.12)	538	(10.73)

		***PE***			
Individual	Own color	5.67	(1.15)	7.66	(1.11)
	Co-actor’s color	4.63	(1.07)	6.25	(1.01)
	Other color	5.06	(0.89)	5.45	(1.12)
Joint	Own color	5.35	(0.71)	8.59	(0.96)
	Co-actor’s color	5.31	(0.72)	6.48	(0.82)
	Other color	6.10	(0.94)	7.55	(0.86)

**Table 4:** The results of ANOVAs on response time and percentage error in Experiment 2.

Factors	*df*	*MSE*	*F*	*p*	$\eta_{\text{p}}^{2}$
	***Response time***				
Task Condition (TC)	**1, 61**	**6,528.23**	**21.04**	**<0.001**	**0.256**
Block Type (BT)	2, 122	3,247.85	2.48	0.088	0.039
Stimulus Compatibility (SC)	**1, 61**	**1,826.00**	**93.26**	**<0.001**	**0.605**
TC × BT	2, 122	1,973.00	2.07	0.130	0.033
TC × SC	1, 61	882.27	<1	0.399	0.012
BT × SC	2, 122	974.52	2.36	0.098	0.037
TC × BT × SC	**2, 122**	**871.25**	**3.63**	**0.029**	**0.056**

	***Percentage error***				
TC	1, 61	79.50	1.41	0.239	0.023
BT	2, 122	32.45	2.64	0.076	0.041
SC	**1, 61**	**28.70**	**17.43**	**<0.001**	**0.222**
TC × BT	2, 122	39.30	<1	0.471	0.012
TC × SC	1, 61	22.63	<1	0.376	0.013
BT × SC	2, 122	26.87	1.77	0.175	0.028
TC × BT × SC	2, 122	27.28	<1	0.609	0.008

*Effects in bold are significant at α = 0.05.*

Response time showed that there were significant main effects of Task Condition (*M*s = 553 ms for the individual task, and 526 ms for the joint task) and Stimulus Compatibility (*M*s = 524 ms for compatible trials, and 555 ms for incompatible trials). There was a significant 3-way interaction among all three variables. To follow up this interaction, Bonferroni-corrected multiple comparisons were performed on the Simon effect in the three blocks separately for the individual and joint tasks. For the individual task, the Stroop effect was 33 ms for the own target color block and 45 ms for the co-actor’s target block, which did not differ significantly (*p* = 0.405). The Stroop effect for the non-target color block (*M* = 19 ms) was significantly smaller than that for the co-actor’s target color block (*p* = 0.031) but not for the own target color block (*p* = 0.277). Therefore, the interaction was driven by the larger Stroop effect for the co-actor’s target block in the individual task phase.

Percentage error showed that the only significant effect was the main effect of Stimulus Compatibility (*M*s = 5.35% for compatible trials, and 7.00% for incompatible trials). The Stroop effects were 1.99%, 1.62%, and.39% for the own target color, co-actor’s target color, and non-target color blocks, respectively, in the individual task, and were 3.24%, 1.17%, and 1.45%, for these three blocks in the joint task.

### Discussion

The results of the present experiment agreed mostly with Experiment 1, except for two outcomes. First, in RT, there was no overall difference in the Stroop effect between the individual and joint tasks. However, in the individual task, the Stroop effect for the co-actor’s target color was elevated as compared to the non-target colors, although the Stroop effect for the own target colors did not differ from the co-actor’s target colors or non-target colors. In the joint task, the Stroop effect was similar among the three types of target colors. That the elevated Stroop effect was obtained only for the co-actor’s target in the present experiment but not in Experiment 1 suggests that it was likely due to the additional requirement to withhold responding when the target color was that of the co-actor’s in the individual task. This may be due to binding of response inhibition with the co-actor’s target colors (e.g., Yamaguchi et al., 2018), which slowed responding when the word meanings were the co-actor’s target colors. Second, in PE, Experiment 1 showed a larger Stroop effect for the own target colors than the co-actor’s target colors or non-target colors, but there were only numerical tendencies (especially in the joint task) but no statistically significant differences in Experiment 2. The lack of this tendency in the individual task might reflect an elevated Stroop effect for the co-actor’s target color as in RT, but the results are not conclusive in this respect. Overall, there was little evidence of semantic gradient across the different types of color words in RT, suggesting that all color words were represented similarly in the individual and joint tasks. There was some tendency in the joint task that the Stroop effect in the own target color block was larger than those in the other blocks, as in Experiment 1.

## General Discussion

The present study used the joint version of the Stroop task and examined whether actors in a joint task setting integrate their co-actors’ part of the task or divide the labor between them. If co-acting individuals share a mental representation of the joint Stroop task, they represent their co-actor’s target colors as if they were their own target colors. Consequently, the co-actor’s target colors should be represented in the same way as the actor’s own target colors, and the Stroop effect would be as large when the color names are from their co-actor’s target colors as when the color names are from their own target colors. If co-acting individuals divide the labor of the Stroop task, the co-actor’s target colors should be represented in the same way as the non-target colors, and the Stroop effect would be smaller when the color names are from their co-actor’s target colors than when the names are from the actor’s own target colors. Both predictions presume the semantic gradient (Klein, 1964); the Stroop effect is smaller for non-target colors than the actor’s own target colors.

Nevertheless, the results of Experiment 1 showed little evidence that the semantic gradient occurred in RT. The Stroop effect for the actor’s own target colors or for the co-actor’s target colors was no different from the Stroop effect for the non-target colors. Given that the semantic gradient has been one of the key findings in Stroop interference, this outcome was unexpected, but the lack of the differences in the Stroop effect for the actor’s own target colors and the co-actor’s target color is consistent with the finding of Saunders et al. (2018). With nearly twice as large sample size as in Experiment 1, the results of Experiment 2 also showed no evidence that the semantic gradient occurred in RT. There were little difference in the Stroop effect between the actor’s own target colors and the co-actor’s target colors. Although the co-actor’s target colors did showed the Stroop effect that was larger than the effect for the non-target colors in the individual task, there is no such evidence in the joint task.

The PE data did show a larger Stroop effect for the actor’s own target colors than for the co-actor’s target colors or the non-target colors in Experiment 1, and the joint task of Experiment 2 also showed this pattern. The outcomes are consistent with the division of labor, but the discrepancy with the RT results made it difficult to consider this finding to be conclusive. Saunders et al. (2018) also found a similar discrepancy between RT and PE and suggested the possibility that the Stroop effect arises from two different processes and the Stroop effects in RT and PE depend on different processes. The present results would be expected if RT mostly reflected stimulus recognition and PE reflected both stimulus recognition and response selection. If so, all distractor words are processed in a similar manner at the level of stimulus recognition, but there is a division of labor at the level of response selection. It should be acknowledged, however, that these results may depend on the use of manual responses because the actors could not make their co-actor’s responses in this setting. It is still possible to utter the co-actor’s target colors if vocal responses are used. Hence, the generalizability of the results to vocal response should be tested in future investigations.

The present results differed from the previous joint Stroop study by Demiral et al. (2016), which suggested that the actors monitored the co-actor’s target colors as reflected in the ERP components during nogo trials that was larger in the joint task than in the individual task. The discrepancy is likely due to the additional task requirement on nogo trials of Demiral et al. (2016) study, in which the actors were required to report whether their co-actor made an error. This requirement forced the actors to monitor and determine the co-actor’s responses on every trial, so co-representation of the co-actor’s trial was built in the task. There was no such requirement in the present study, so the actors were free to choose whether to represent their co-actor’s target colors. The present results suggest that the actors did not choose to represent their co-actor’s target colors.

This conclusion corroborates the recent findings from other types of joint tasks. For instance, co-acting individuals in the joint Simon task monitored the proportion of compatible trials of their own but did not monitor the proportion of compatible trials of their co-actor’s (Yamaguchi et al., 2018). Although the actors appear to monitor certain information about stimuli to which their co-actor responded in the joint Simon task, it is simply because the stimulus information is required to determine whether the actors had to make response on that trial (e.g., colors) or because encoding of the stimulus information was obligatory (e.g., stimulus location; Treisman and Gelade, 1980; Logan, 1998). Thus, representing certain aspects of the co-actor’s stimuli was also built in the task itself. It has also been argued that certain aspects of the co-actor are salient and may be used as a reference point to represent part of the actor’s own task (Dolk et al., 2014; Prinz, 2015), which would explain why the Simon effect is obtained in the joint task while the proportions of compatible and incompatible trials for an actor does not affect the Simon effect on the other actor. Similarly, a study using a joint version of task switching also showed switch cost was obtained only when the preceding trial was performed by the same actor as the current trial, but not when the preceding trial was performed by the co-actor (Yamaguchi et al., 2017b). This finding also suggests that the actors recognize the task on a preceding trial as their own if they actually performed the trial for themselves but not if their co-actor performed it, implying the division of labor.

There is a study showing that actors recognized stimuli presented to their co-actor better than stimuli that were new (Eskenazi et al., 2013), suggesting that they do not ignore irrelevant stimuli assigned to their co-actor. The present study does agree that the actors processed the color names from the co-actor’s target colors to a degree that the co-actor’s colors still produced the Stroop effect, but there were no significant differences between the co-actor’s colors and the non-target colors and no evidence that the co-actor’s color names were processed so far as to activate the action program representing the co-actor’s response (Knoblich and Sebanz, 2006; Sebanz et al., 2006). Therefore, the co-actor’s part of the task appears to have no special status in the present task setting.

Although a strong claim about task co-representation receives little support from the present results, it is clear that joint task would benefit from both the division of labor and the integration of the co-actor’s part of the joint task into one’s task representation, but the question of which part of the co-actor’s task is taken into account in a joint task setting still remains unanswered. There are at least two possibilities by which the division of labor and the integration of task representation coexist in a joint task setting. The first possibility is that they are two different modes of joint task performance from which actors can choose depending on the demands of the given task setting. For instance, there are a number of studies on the joint Simon task that showed that the joint Simon effect depended on various social factors (e.g., Hommel et al., 2009; Ruys and Aarts, 2010; Iani et al., 2011). It has also been shown in joint task switching that switch cost was reinstated after the co-actor’s trials when two actors shared the same action effect (Yamaguchi et al., 2017a). These observations seem to be consistent with the two-mode hypothesis of the joint task performance.

The second possibility is that the integration of task representation and the division of labor reflect two different levels of cognitive processes that control joint task performance. Cognitive processes are structured hierarchically (e.g., Logan and Crump, 2011), with the higher level process monitoring information relevant to the global task goal and the lower level process monitoring information relevant to the local task goal. In a joint task setting, the higher level process may monitor aspects of the task that are relevant to the global goal such as who performs a given trial, while the lower level process monitors aspects of the task that are relevant to the local goal such as which of the alternative responses should be made. It is possible that the higher level process represents aspects of the co-actor’s part of the task as long as it is relevant to the global goal of selecting an actor.

The hierarchical processing hypothesis is consistent with recent findings from a naming task (Philipp and Prinz, 2010), in which actors uttered their own name or their co-actor’s name in response to target stimuli (black and white diamonds) that were superimposed on a photographic image of the actor, the co-actor, or an unfamiliar individual. A critical finding was that, in the joint task for which each participant uttered only one of the names in response to one of the targets, responses were faster when the picture was the actor’s own face than when it was their co-actor’s or that of an unfamiliar individual (the face-actor compatibility effect; also see Baess and Prinz, 2017), but responses did not depend on whether the name was compatible with the picture (the face-name compatibility effect). When a single actor performed the same task alone with the two alternative names, the face-name compatibility effect emerged. The authors suggested that the irrelevant pictures primed who to take a turn on a given trial when two responses are divided between two actors in the joint task, but the same pictures primed what response should be made when the two responses are assigned to a single actor in the individual task. Wenke et al. (2011) also proposed that actors in joint task settings represent when it is their own turn or the co-actor’s turn, rather than the actions that co-acting individuals perform. Therefore, joint performance reflects conflict in actor identification, but not conflict in response selection.

There are not enough data to distinguish between these two possible mechanisms of joint performance in which the integration and division may co-exist. Further investigations are necessary to explore what cognitive mechanisms support the division of labor and the integration of task representations. As a general remark, theories of group cognition and team performance tend to emphasize the similarity of individuals as a hallmark of collective behavior (Wegner, 1986), but an advantage of group performance also comes from the diversity of knowledge and skills (Lewis and Herndon, 2011). Studies of joint performance has focused mainly on what is shared between co-actors, but limited attention has been paid to what is divided between co-actors (Wahn et al., 2017). These two questions serve two ends of the spectrum in task sharing. Future studies of joint task performance should shed light on the processes and representations that underlie effective task sharing by assessing how individuals balance the divide of the labor and the integration of task representations in a joint task setting.

## Author Contributions

MY conceived, designed, and prepared the materials. MY supervised data collection of Experiment 1, and EC and DE collected the data of Experiment 2. MY wrote the first draft. All authors contributed to revisions and approved the final version.

## Conflict of Interest Statement

The authors declare that the research was conducted in the absence of any commercial or financial relationships that could be construed as a potential conflict of interest. The reviewer PS andhandling Editor declared their shared affiliation at the time of the review.
